# Advancing Diabetes Prevention and Control in American Indians and Alaska Natives

**DOI:** 10.1146/annurev-publhealth-093019-010011

**Published:** 2022-04-05

**Authors:** Julie E. Lucero, Yvette Roubideaux

**Affiliations:** 1 Department of Health and Kinesiology, College of Health, University of Utah, Salt Lake City, Utah, USA; 2 Policy Research Center, National Congress of American Indians, Washington, DC, USA

**Keywords:** type 2 diabetes mellitus, American Indian, Alaska Native, prevention and control

## Abstract

As with many Indigenous populations globally, American Indians and Alaska Natives (AI/ANs) experience high rates of type 2 diabetes. Prevention efforts, ongoing medical care, patient self-management education, and support to prevent and reduce the risk of long-term complications must be developed to limit the impact of diabetes on individuals, families, and communities. Diabetes prevention and control require both individual- and community-level efforts as well as policies that attempt to mitigate contributing adverse socioeconomic factors. Congressional funding since 1998 continues to address the epidemic of diabetes in AI/AN groups with the Special Diabetes Program for Indians (SDPI), which has resulted in significant outcomes and key lessons that can inform new efforts to prevent diabetes in other populations and communities. The purpose of this review is to understand the context behind the epidemic of diabetes in AI/ANs, review the impact of the SDPI on prevention and control of diabetes as well as the translation of these strategies into clinical practice and their influence on health practice, and identify lessons learned for future efforts to address this ongoing challenge for AI/AN and other communities suffering from type 2 diabetes.

## INTRODUCTION

Health disparities affecting American Indian/Alaska Native (AI/AN) communities in the United States are well documented and contribute to a life expectancy that is 5.5 years lower compared with that of other Americans. One example of a health disparity is type 2 diabetes mellitus (diabetes), which disproportionately affects AI/ANs as well as many Indigenous populations globally (15). According to the Indian Health Service (IHS), age-adjusted diabetes mortality rates in these communities are 3.2 times greater than those of the US all races population (19). Diabetes affects how the body uses blood sugar and, if untreated or uncontrolled, can cause disabling conditions and contribute to more severe disease, such as cardiovascular disease, end-stage renal disease, or even death. Because it is a chronic condition, diabetes requires ongoing medical care, patient self-management education, and support to prevent and reduce the risk of long-term complications. While clinical care tends to focus on individual behaviors, social and environmental factors play a prominent role in the prevention and control of diabetes (6).

The social ecological context for addressing diabetes in AI/ANs has been an important consideration as interventions have been developed over the years, given the historical/political origins and social determinants of risk factors for diabetes in this population. The purpose of this review is to provide an update on, and discuss the context behind, the epidemic of diabetes in AI/ANs and identify the impacts of the Special Diabetes Program for Indians (SDPI), which offers multilevel interventions that address individual- and community-level factors. It is necessary to examine the translation of the clinical findings of the SDPI into interventions that improve the health of individuals, families, and the public by identifying lessons learned for future prevention and control efforts.

## EPIDEMIOLOGIC CONTEXT OF DIABETES AMONG AI/AN POPULATIONS

AI/ANs persistently experience the highest prevalence of diabetes among all US racial/ethnic groups (1, 3). In 2017 IHS data, the age-adjusted prevalence of diagnosed diabetes was 14.5% for AI/AN men older than 18 years and 14.8% for women. Age-adjusted prevalence of diagnosed diabetes for AI/ANs regardless of sex (14.7%) was higher compared with people of Hispanic origin (12.5%), non-Hispanic blacks (11.7%), non-Hispanic Asians (9.2%), and non-Hispanic whites (7.5%) (3). Even though these prevalence rates from IHS data include only those who receive care in IHS facilities, estimates from the Department of Veterans Affairs clinical database reveal similar results for AI/AN patients (30).The prevalence of diabetes in AI/AN populations varies by region, with the highest rates shown in the IHS Southwest Region at 21.1% in 2017 (1). Although this disparity has persisted for several decades with increasing trends, recent IHS data reveal that the prevalence of diabetes in AI/AN adults decreased from 15.4% to 14.6% from 2013 to 2017 (1). Even though the prevalence rate has decreased, diabetes remains the fourth leading cause of death for AI/ANs (19).

Exacerbating the problem of diabetes in AI/AN individuals are complications and the occurrence of comorbidities (the occurrence of two or more chronic conditions in the same person). To quantify comorbidities in the general population, Iglay et al. (16) found that among people diagnosed with diabetes, 97.5% had at least one, and 88.5% had at least two, comorbid conditions such as obesity, cardiovascular disease (CVD),and sleep disturbances. Such comorbidities are seen with type 2 diabetes in AI/AN populations. Researchers using IHS data found that obesity was present in 69.6% of AI/AN patients 18 years and older with diabetes (48). Diabetes is a well-known risk factor for CVD, which is the leading cause of death in the United States and in AI/ANs (19). AI/ANs have the second highest rates of CVD mortality at 74 and 161 deaths per 100,000 among women and men, respectively (5). Living with diabetes and at least one comorbidity can limit daily living activities. Goins et al. (14) found that among AI elders, 24% reported having three or more limitations, compared with 3% among the White participants. The burden of other morbidities on AI/AN individuals with diabetes in one study was found to exceed that of insured US adults with diabetes by 50% (33).

## HISTORICAL AND POLITICAL CONTEXTS OF AI/ANs

Although diabetes and comorbidities are widespread in this moment, history tells a different story. As described elsewhere (36, 45), the documentation of the diabetes epidemic in AI/ANs became clear in the early 1970s. The National Institutes of Health (NIH) studies with the Pima Indians (now the Gila River Indian Community) found higher rates of diabetes in this group than in the general US population, and both genetic and environmental factors were found to contribute to this disparity (32). However, West (45) posits that diabetes was most likely uncommon in Native populations prior to the NIH studies. While studies that focused on the Pima Indians of southern Arizona provided some evidence of increased genetic risk, environmental or acquired factors have played a significant role in the diabetes epidemic (36, 45), especially since genetic variance may explain only ~5–10% of the risk for type 2 diabetes (11).

Among the most challenging parts of the epidemic’s genesis in AI/ANs is that it likely occurred as a result of significant and forced changes in lifestyle and environmental factors (32) that to this day are difficult to overcome. The US political history involving AI/ANs maps out the social insults that have contributed to health disparities. The disruption of traditional ways of life began with early US policies such as relocation, boarding schools, and underfunding of health services for AI/ANs. Shelton’s (38) comprehensive timeline of the historical and legal bases for AI/AN health care begins with the Doctrine of Discovery decision by the US Supreme Court in 1823, which affirmed the authority of European/Christian settlers to remove Native inhabitants from lands either by purchase or conquest. The Doctrine of Discovery has had a lasting effect in Supreme Court decisions that have undermined the sovereignty and rights of AIs (31).

For more than 2,000 years, the Pima Indians subsisted through irrigation farming in their desert environment. However, new settlers in the late nineteenth century disabled the irrigation systems, disrupting Pima Indians’ inability to farm and changing their way of life. The inability to farm caused a reliance on government surplus commodities with high carbohydrates and fatty foods, which, combined with the reduced physical activity, likely contributed to the increased prevalence of obesity and diabetes (36). This story is not unique to the Pima Indians. The relocation and disruption of traditional lifestyles were repetitive patterns across AI/AN Tribal Nations and no doubt contributed to an increase in diabetes incidence and prevalence.

As traditional lifestyles were replaced by Western lifestyles, diabetes risk was also aggravated by the sociopolitical cultural contexts. Occurring simultaneously was the egregious relocation of AI children to boarding schools. Between 1877 and 1926, AI children were forcefully removed from their families and sent to boarding schools, where Christian values were rooted. As Christian values were introduced, anything representing Native cultures was suppressed to “kill the Indian in him, and save the man” (37, p. 46). Furthermore, according to Shelton (38), children were subjected to personal violence and not allowed to see their parents or other family members for long periods of time, sometimes years. Today we call these traumatic experiences adverse childhood experiences (ACEs), which are shown to disrupt healthy brain development, affect social development, compromise immune systems, lead to substance misuse and other unhealthy coping behaviors throughout the life span, and impede parenting capacity (12). The impact of ACEs on parenting ability produces intergenerational trauma, whereby the trauma experienced transcends generations and creates a legacy of trauma (8) that may be reflected in the high burden of behavioral health conditions in AI/ANs in the context of their need for more health care and behavioral health services (32).

As a result of relocation and land allocation, the US government entered into treaties that included various promises to provide health care for AI/AN Tribal Nations. Since the nineteenth century, minimal services were provided through the Bureau of Indian Affairs (BIA) in the Department of the Interior (27). In 1921, the US Congress passed the Snyder Act to authorize funding for the “relief of distress and conservation of health” in Indian communities (38, p. 18). After several reorganization attempts, the 1954 Transfer Act assigned to the US Public Health Service the responsibility for AI health services, and the IHS was created (26, 38) to provide health care services on or near AI reservations. Today, the IHS provides mainly primary care and some referral care in a system of hospitals, clinics, and health stations located on or near AI/AN reservations. With passage of the Indian Self-Determination and Educational Assistance Act of 1975 (Pub. L. 93–638, 88 Stat.2203,42 U.S.C. §§ 450–458), health programs previously managed by the IHS were allowed to be managed by Tribes. However, the IHS has yet to be adequately funded, and thus many disparities persist (19). For an in-depth review of the dynamic changes experienced by the Indian Health Service, see Kruse et al. (26) in this *Annual Review of Public Health* volume.

## SOCIAL ECOLOGICAL STRATEGIES FOR AI/AN DIABETES PREVENTION

**Figure 1:**
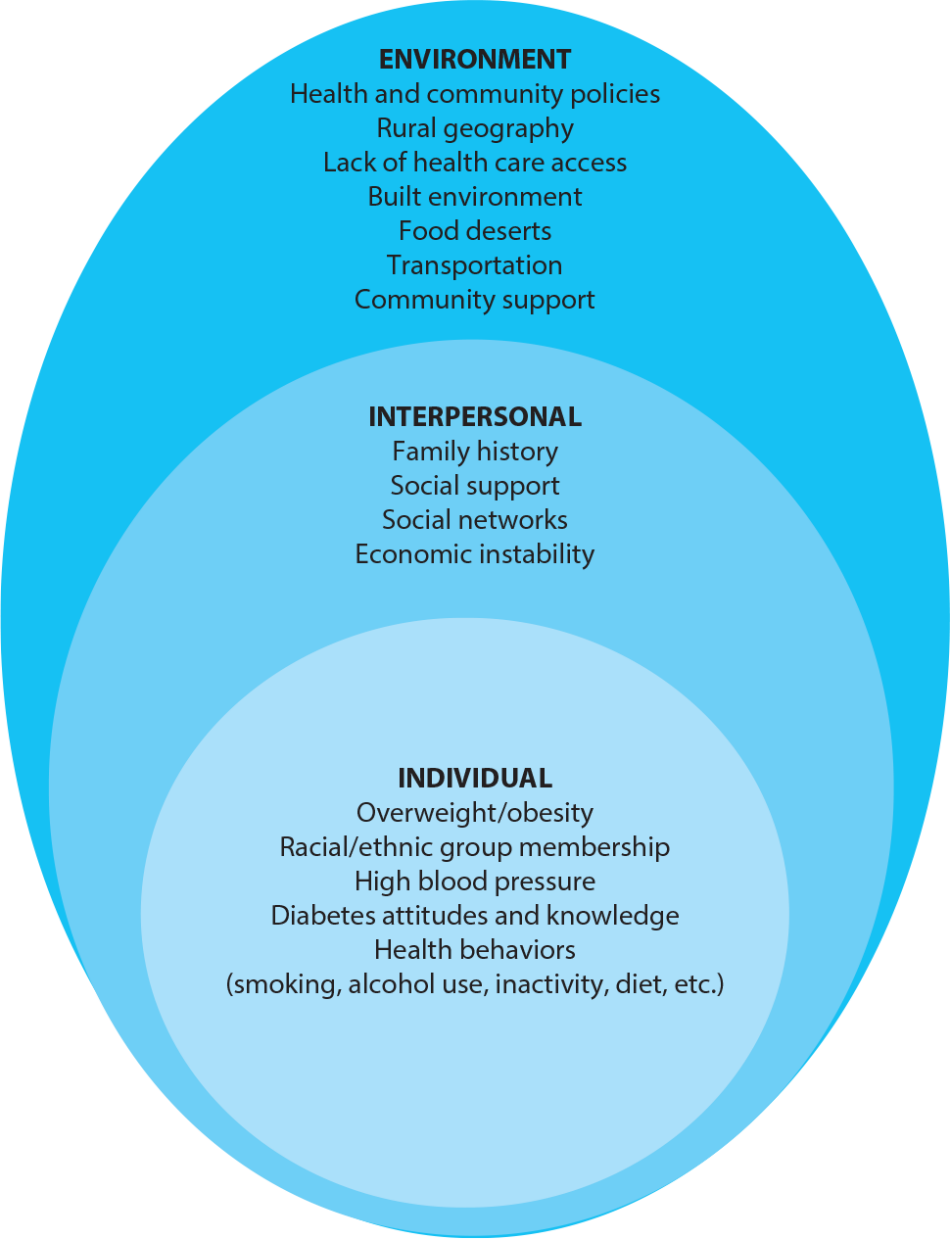
Social ecological framework describing diabetes risk factors grouped by framework level.

As a result of the historical and political contexts, solutions for preventing and controlling diabetes require a multifaceted or ecological approach. Many national and global research initiatives have focused on identifying social determinants that either protect against or create more risk for developing diabetes and its complications (15). For example, research acknowledges that the burden of diabetes and other health disparities is rooted in the intergenerational traumas as well as in poverty and poor social conditions (46). Specific to diabetes self-management, Clark & Utz (6) identified the built environment, economic stability, education, health care, and social and community support as social determinants that must be part of improving diabetes outcomes, a recommendation shared by health disparities researchers. To help organize social determinants, ecological models provide a framework to categorize factors that contribute to diabetes disparities in AI/AN communities. The social ecological model, a common ecological framework, recognizes multiple levels within a social system and how interaction between levels correlates within a system (29).Figure 1 provides a summary of influential diabetes risk factors according to three levels of an adapted social ecological model—individual, interpersonal, and environmental—that can be addressed through interventions to prevent and treat diabetes. The environmental level includes community, organizational, and policy levels.

Most attention in diabetes research is given to risk factors at the individual level. Risk factors at this level include individual characteristics, such as demographics, as well as knowledge, behavior, and attitudes about diabetes. The Centers for Disease Control and Prevention (CDC) reports a higher diabetes risk for individuals who are overweight or have obesity, have a family history of diabetes, are physically inactive, have high blood pressure, or currently smoke or who are in certain racial/ethnic populations (African American, Hispanic/Latino, and/or AI/AN) (4). Using population data, Cobb et al. (7) found that 42% of male and 32% of female AI/AN respondents reported being overweight [body mass index (BMI) = 25×29.9 kg/m^2^] and 27% male and 32% female respondents reported no leisure time physical activity. In the same study, 31% of males and 28% of females in the AI/AN sample reported being told that they had high blood pressure. Regarding tobacco use, 34% of male and 30% of female AI/AN respondents reported being a current smoker (7). These data illustrate the proportions of the AI/AN population that have at least one individual-level diabetes-related risk factor.

The interpersonal level of the social ecological model includes both formal and informal social networks and support systems, as well as a family history of diabetes, household economic instability/poverty, and intergenerational trauma. Social support or perceived support is a well-accepted predictor of diabetes self-management, which is a dominant predictor of glycemic control for type 2 diabetes (40). According to the 2018 American Community Survey, ~25% of AI/ANs across the United States live in poverty, which is the highest poverty rate by race (35).

Figure 1 illustrates how, in general, the environmental level of the social ecological model is embedded in larger social, cultural, and economic structures that have a cumulative effect on health (39). Diabetes-related environmental-level risk factors include health and community policies such as school policies, rural geography, lack of access to medical care, the built environment including food deserts, community support, and economic and noneconomic infrastructure such as public transportation. To illustrate the interaction between environmental risk factors and diabetes, tribal lands are often located in rural geography and within areas that are short on health care and mental health care providers. Transportation (part of the built environment) in rural communities is a widespread barrier to accessing medical services and healthy foods (6). In one study, over half of respondents were required to travel more than 20 miles round trip to shop for food (28).

Compounding the transportation issue is underfunding of medical care. According to a 2018 report by the US Commission on Civil Rights (43), the IHS spent $3,332 per person in 2017 for health care and other programs, including preventive care, compared with $9,207 per person for nationwide health care spending. Because an estimated one-quarter of people with diabetes in the United States may be undiagnosed (3), and early identification is needed for control and prevention of complications (44), access to affordable health services is needed to address the diabetes epidemic. However, the 2010 US Census found that 78% of AI/ANs live in areas that are not defined as tribal statistical areas (34) and may not be eligible for or have access to IHS services, such as the SDPI, if they do not live in an urban area with an IHS-funded urban Indian health program.

Across the social ecological model, risk factors contribute to the high rates of diabetes in AI/AN populations compared with other US racial groups. Having a comprehensive understanding of these risk factors at multiple levels provides an opportunity to develop more effective prevention and control interventions. Health researchers have become increasingly interested in developing and implementing multilevel interventions with the expectation that substantial and sustained change is achievable by prioritizing sources of influence at multiple levels (42).

## THE SPECIAL DIABETES PROGRAM FOR INDIANS

The Special Diabetes Program for Indians (SDPI) is an excellent example of a multilevel intervention for diabetes treatment and prevention. This program was established by Congress in 1997 as a grant program for the prevention and treatment of diabetes in AI/AN communities. With oversight from the IHS, the SDPI initially focused on community-led interventions to treat diabetes and prevent complications that were adapted to local needs in more than 300 IHS, tribal, and urban Indian health programs (SDPI community-directed programs). The SDPI overall was designed to be community-driven, and interventions were based on best practices and adapted to the local community needs.

The SDPI significantly increased access to a wide variety of quality diabetes care practices and services that resulted in improved clinical outcomes in people with diabetes (47). Table 1 describes some improvements in access to diabetes services such as diabetes clinical teams, patient registries, nutrition services, and culturally tailored diabetes education materials since the beginning of the SDPI.

The diabetes prevention and treatment services were provided by the grant programs from a menu of best practices in diabetes care (21). The SDPI community-directed programs also implemented a wide variety of diabetes prevention and treatment programs and services relevant to the local communities. Such programs included community exercise classes and walking/running programs; traditional AI/AN food and nutrition activities, including cooking, classes, and gardening; establishment of tribal wellness policies; group support and individual weight management programs; culturally appropriate diabetes education programs; and partnerships with schools, businesses, and community programs. The primary focus of these programs was health promotion within the community. However, some participating programs reported having guidelines, policies, or campaigns to limit screen time (35% of programs) and school-based nutrition services to meet current nutritional guidelines (59%) for children and youth (18). However, assessment of the effectiveness of the SDPI community-directed programs on diabetes was not conducted as part of the SDPI evaluation because each community adopted their own community-based solutions. The IHS evaluated the SDPI program with grant program reports and monitored outcomes through the IHS and other federal sources of data, including the IHS Diabetes Care and Outcomes Audit, which tracks selected diabetes care measures annually (17).

In 2002, Congress authorized additional SDPI funding for two demonstration programs focused on translating the latest research into the prevention of diabetes and CVD, a major complication of diabetes, in AI/AN communities. The SDPI Diabetes Prevention (SDPI DP) and the SDPI Healthy Heart (SDPI HH) demonstration programs were developed as a competitive grant program for IHS, tribal, and urban Indian programs. Starting in 2004, 66 demonstration programs were funded (36 programs in the SDPI DP and 30 programs in the SDPI HH). Congress required an intensive evaluation of these programs, which are examples of multilevel interventions that were successfully implemented (18).

The SDPI DP program goal was to prevent the development of diabetes among AI/AN individuals who met criteria for prediabetes. Beginning in 2004, 36 IHS, tribal, and urban Indian health program sites participated in the SDPI DP, serving 80 tribes in 18 states and 11 IHS administrative areas. The SDPI DP delivered an adapted version of the evidence-based Lifestyle Balance program developed by the Diabetes Prevention Program (DPP) Lifestyle Resource Core at the University of Pittsburgh Medical Center (9). The adapted version of the curriculum contained 16 sessions delivered within a group setting rather than to individual patients, as was done in the original NIH DPP. Five sessions addressed healthy food choices and food preparation techniques to reduce fat and calorie intake, and 11 sessions covered physical activity, stress management, and self-motivation. A health educator or dietitian delivered the education sessions.

The SDPI DP was implemented with two distinct phases: an initial intensive curricular phase followed by a maintenance phase. The maintenance phase reinforced the DPP curriculum with one-on-one case management lifestyle coaching to determine participants’ needs and goals, develop individualized nutrition and physical activity plans, and help identify and solve participation barriers. Participating programs were also encouraged to develop recruitment, curricular adaptations, and community-based activities based on local culture and community needs in addition to a set of core elements that all programs implemented for the diabetes prevention intervention.

The goal of the SDPI DP was to achieve and maintain at least a 7% weight loss through nutrition and exercise modification coupled with healthy lifestyle education. This degree of weight loss as a program goal was used in the original DPP research trial (9). The intervention included three assessments: baseline survey and medical assessment; postcurriculum assessment; and one-year follow-up.

At baseline, 74% of participants were female, 59% were under 50 years of age, 60% had some college education or higher, 71% were employed, and 60% were married or living as married. Despite the high level of education among participants, only 28% reported an annual household income of $50,000 or higher (23). The SDPI DP evaluation reviewed outcomes along with various factors that influenced those outcomes, and the results to date provide evidence on how this multilevel intervention in a diverse set of programs successfully reduced the incidence of diabetes in those at risk and identified multilevel factors associated with outcomes.

## IHS REPORTED TRENDS IN DIABETES CLINICAL OUTCOMES

**Figure 2:**
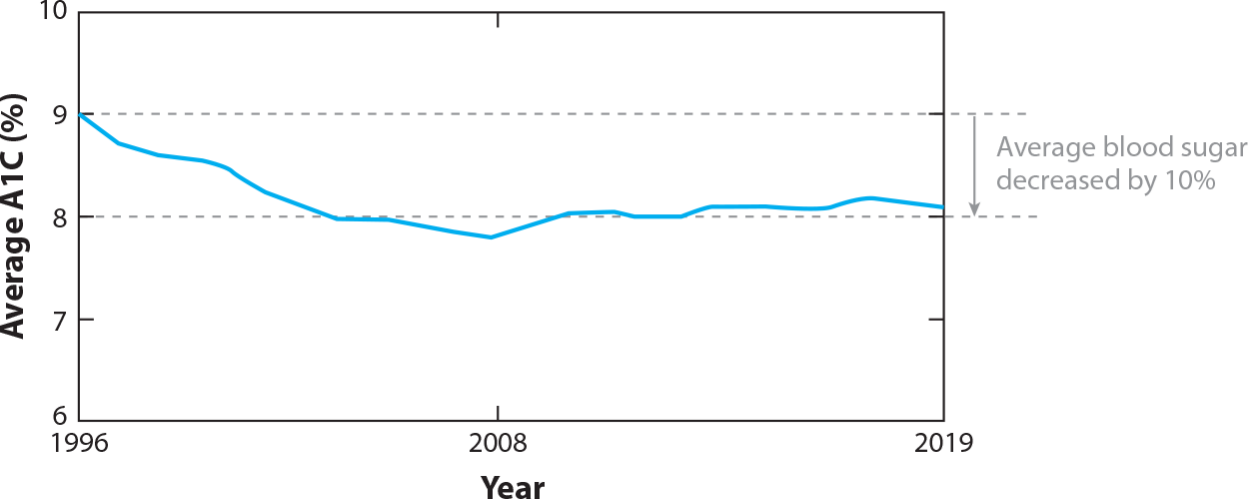
Average glycated hemoglobin (A1C) in patients with diabetes in the Indian Health Service (IHS), 1996–2019. Figure adapted from the Special Diabetes Program for Indians 2020 Report to Congress (20).

Over the same time period as the SDPI, the IHS reported improvements in short-term clinical outcome measures for AI/AN people with diabetes: Average A1C has decreased by 10%, average low-density lipoprotein (LDL) cholesterol has decreased by 24%, average blood pressure has remained in a well-controlled range, and current smoking use has recently declined. Figure 2 illustrates the decline in average A1C during the same time period as the SDPI as reported by the IHS (20).

**Figure 3:**
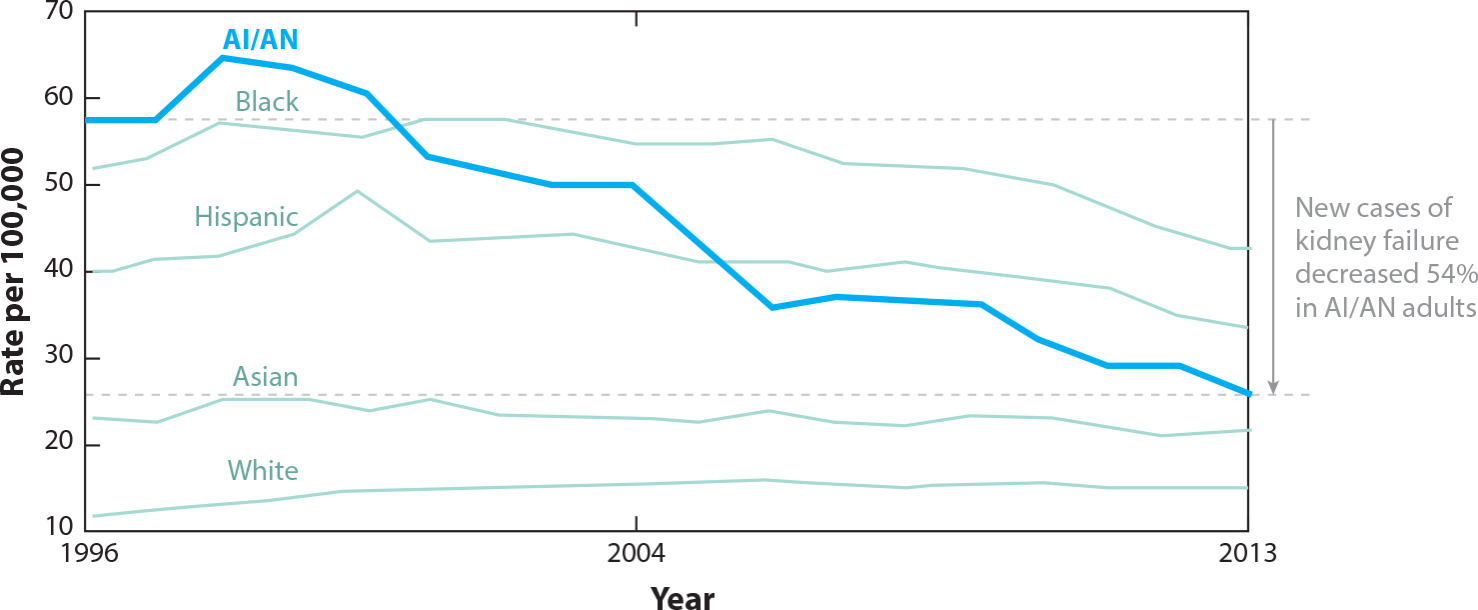
Incidence of diabetes-related kidney failure in US adults. Abbreviation: AI/AN, American Indian and Alaska Native. Figure adapted from the Special Diabetes Program for Indians 2020 Report to Congress (20).

Improvements in blood glucose have resulted in reduced diabetes complications (13), and the SDPI grantee activities that focused on prevention and enhanced treatment of diabetes likely contributed to improved long-term outcomes. The IHS reports that hospitalizations for uncontrolled diabetes in AI/AN adults and diabetes-related mortality in AI/ANs are now decreasing. The IHS also reports a 50% reduction in diabetic retinopathy over the time period of the SDPI (2). The most notable outcome over the past 20 years has been the 54% decrease in new cases of diabetes-related kidney failure in AI/AN adults, which is faster than the rate of decrease among other racial and ethnic groups (20) (Figure 3).The IHS recently reported that the prevalence of diabetes in AI/AN adults is now decreasing after decades of an increasing trend, and this decrease began during the time of the SDPI (1). Although these improvements may have been due in part to secular trends, many believe the additional funding and efforts of the SDPI played a significant role in these improved outcomes.

## OUTCOMES OF THE SDPI DP PROGRAM

The IHS clinical outcomes described above are for the total IHS population during the same period as the SDPI overall initiative. This section reviews outcomes directly related to the SDPI DP evaluation. Over a 10-year period, 8,652 AI/AN individuals enrolled into the SDPI DP and ~5,624 (65%) completed a postcurriculum assessment. During postintervention follow-up, significant changes were reported in participant weight loss. After completing the SDPI DP curriculum, among those with postprogram weight measures, 36% lost more than 5% of their initial weight, 17% lost 3–5% of their weight, and 47% did not achieve a weight loss of more than or equal to 3% (24). More than half of those who completed a postcurriculum assessment lost and maintained at least 3% of their weight loss.

**Figure 4:**
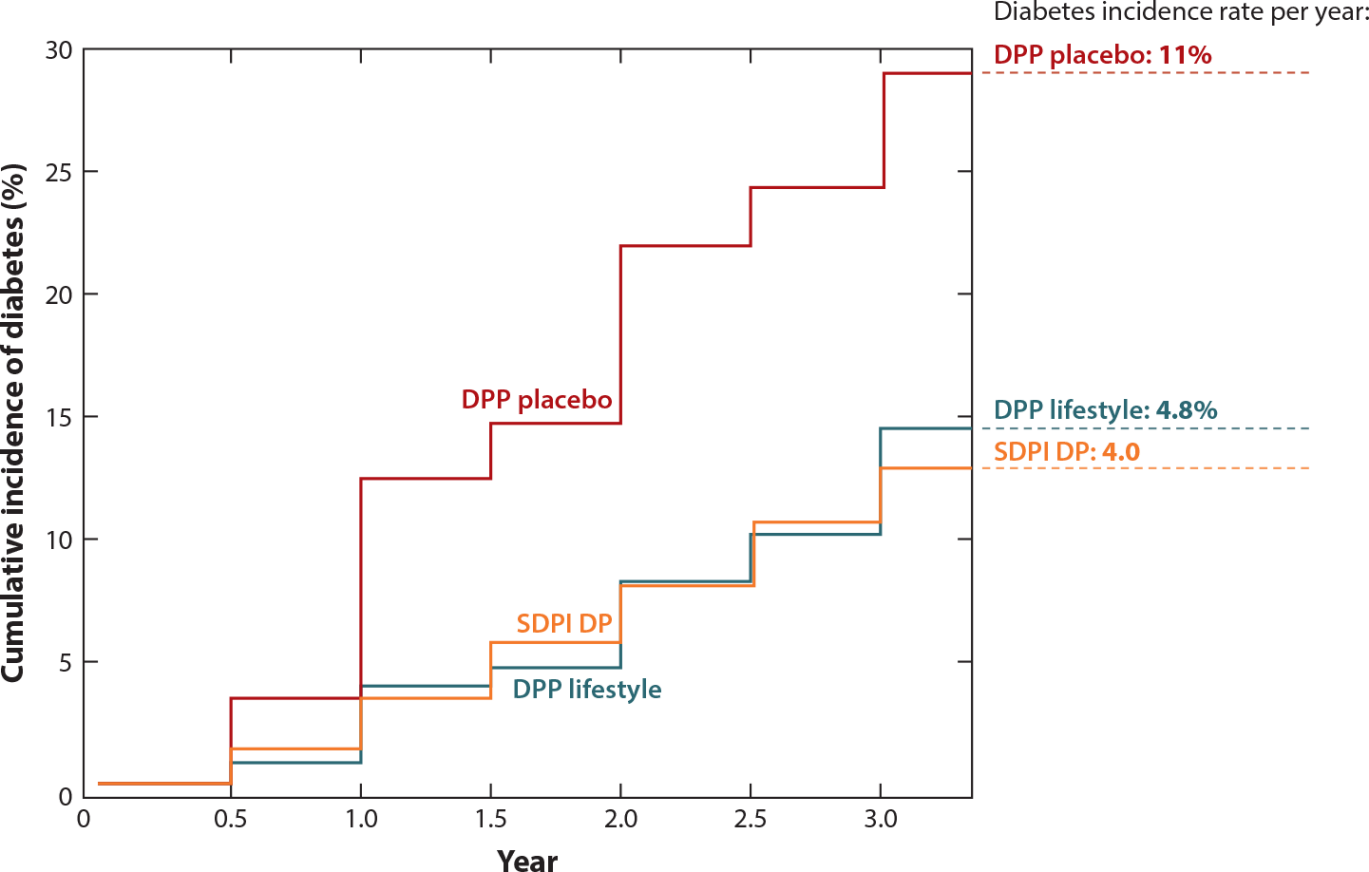
SDPI DP cumulative incidence of diabetes at three years after lifestyle intervention. The figure is a visual comparison with the NIH DPP. Results of the NIH DPP and the SDPI DP are superimposed in the graph for comparison, but participant characteristics and study design were not identical. Abbreviations: DPP, Diabetes Prevention Program; NIH, National Institutes of Health; SDPI DP, Special Diabetes Program for Indians Diabetes Prevention (program). Figure adapted from the SDPI 2011 Report to Congress (18).

Slowing the onset of diabetes in participants who were at risk for the disease was the primary outcome of the SDPI DP program. Of the 2,553 participants who enrolled by July 2008, 74% who completed all 16 sessions had a significantly lower incidence of a diabetes diagnosis, with a crude rate of 3.5% among completers compared with noncompleters at 7.5% (25). Participants who completed the SDPI DP program were older, were more educated, had a higher household income, were retired, or were employed and had lower baseline weight and fasting blood glucose levels. About 23% of program completers met the 7% weight loss goal and increased physical activity by 82 min per week after completing the program lessons. The diabetes incidence rate for participants in the SDPI DP was lower than the incidence rate in the NIH DPP placebo group (11%) and similar to that of the AI/AN participants in the NIH DPP lifestyle intervention group (4.8% per year) (18) (Figure 4).

In the first 6 years of follow-up for the SDPI DP, participants who had lost more than 5% of their initial weight had a 64% lower risk of developing diabetes, and those with 3–5% weight loss had a 40% lower risk compared with those who did not achieve at least 3% weight loss. After year 6, the group with greater than 5% weight loss had a 38% lower risk of developing diabetes compared with the 3% weight loss group, but its diabetes risk was not significantly different from that of the group with 3–5% weight loss (24).

## FACTORS ASSOCIATED WITH WEIGHT LOSS

Weight loss was a key to diabetes control and outcomes of the SDPI intervention. Because the educational curriculum provided lifestyle coaching sessions and community-based exercise programs, program data from SDPI participants were used to understand the most influential factors associated with weight loss, a primary outcome of the lifestyle intervention. Dill et al. (10) explored the influence of psychosocial factors, Jiang et al. (22) looked at socioeconomic factors, and Teufel-Shone et al. concentrated on changes to diet (41). Over the course of the intervention, Dill et al. (10) reported that weight loss was lower when psychological distress and negative family support were higher. The ability to cope and spirituality were associated with more weight loss.

Jiang and colleagues (22) found that weight loss was significantly higher among male, older, retired, and married participants. The authors also looked at physical activity, finding that those with an education level less than high school and baseline annual household income of less than $15,000 reported fewer gains in physical activity compared with other participants. Participants reporting lower household income had a smaller reduction in BMI, less physical activity, and less healthy diets from baseline to post assessment. Teufel-Shone et al. (41) found that participants in the SDPIDP who were young males, had low income, or less education more frequently consumed unhealthy foods, but consumption did not vary by urban or rural setting.

In another study focused on participant attrition, older female participants had significantly decreased risk for both short- and long-term attrition. Those with increased risk of attrition had lower household income, no family support person, and more chronic pain. Sites categorized as medium (5,000–9,999 user population) graduated more participants from the 16-week curriculum compared with large sites (>10,000). Furthermore, younger staff (<40 years) and increased reporting of participation barriers also resulted in more long-term attrition (25a). Using multivariate logistic regression, seven independent variables were identified as predictors for program attrition. These predictor characteristics included being male, being between the ages of 18 and 60 (2 categories), earning less than $30,000 annually (two categories), reporting more than two comorbid conditions, and rating general pain at more than 4 on a visual index. For long-term attrition, 5 risk factors were identified: being male; being between the ages of 18 and 60 (2 categories); being separated, widowed, or divorced; and rating general pain at more than 4 on a visual index. In terms of site characteristics, predictors with the largest impact on program attrition were small- or large-site user population and young staff member age (<40 years old).

The findings of the evaluation of the SDPI published so far by the IHS and in the peer-reviewed literature associate a variety of demographic, health, social, economic, and community factors with better or worse outcomes, with relatively consistent findings indicating that participants impacted negatively by these factors were not among those with the best outcomes.

## CONCLUDING REMARKS

The SDPI program’s impact on the prevention and treatment of diabetes in AI/AN communities is clear, and the evaluation of this program over the two-decade intervention has helped investigators track and understand outcomes. The vast diversity of the 574 federally recognized tribes in the United States and their status as sovereign nations present unique challenges to conducting diabetes prevention and treatment interventions in AI/AN communities. With more than 300 distinct cultures and traditions represented in tribal communities, designing interventions requires the ability to adapt interventions to meet the unique needs of each AI/AN community and to address the unique challenges and potential barriers to success. Examining the epidemiology, the historical and political contexts, and the social ecological factors that may impact the success of these types of interventions is helpful to understanding which types of interventions can be more successful at preventing diabetes in AI/AN communities. Although the SDPI program did provide many resources at the intrapersonal, interpersonal, organizational, and community levels, more work at the policy level (i.e., changes to the built environment, taxes on sugar-sweetened beverages) is needed. Given that weight loss and healthy weight are major factors for diabetes risk reduction, policy that assists this effort is paramount, especially with youth, as health behaviors are developed in early life stages. This area requires future research and demonstration in AI/AN communities to improve diabetes incidence and management.

This review has focused on the SDPI and its SDPI DP demonstration program and the efforts to implement diabetes prevention and treatment programs in real-world, diverse AI/AN communities. The SDPI began with community-directed programs, with a focus on best practices to prevent and treat diabetes in the more than 300 diverse grantees, and these efforts increased access to quality diabetes services and improved outcomes over time. Fortunately, the IHS already had in place its IHS Diabetes Care and Outcomes Audit, along with other IHS clinical data to monitor and track outcomes for participating IHS, tribal, and urban Indian health care programs. This ability to monitor data over time helped ensure and understand the program’s success.

The SDPI and the SDPI DP were multilevel interventions that included individual, health system, and community strategies that were adapted to the local community and resulted in positive outcomes that were impacted by a variety of factors, including many social determinants of health. The willingness of program administrators and staff to participate in this unique demonstration project along with the collaborative evaluation process allowed for an assessment of outcomes and of many different factors associated with those outcomes, all of which have served to inform future interventions.

While a significant commitment of funding and time was required to design and implement the SDPI initiative, the evaluation results, reports, and subsequent publications have provided important knowledge and lessons learned for the development of similar programs in other communities. The positive evaluation and outcomes of the SDPI indicate the importance of developing diabetes prevention programs that include strategies at multiple social ecological levels. Although Congress required the SDPI to include an evaluation of its impact as an effort to translate research into practice, an evaluation of community-based program and policy initiatives is also needed in future work. A holistic evaluation will allow diabetes researchers to understand the SDPI ripple effects and system changes that can contribute to diabetes prevention and control in AI/AN and other communities.

Since the landmark results of the NIH-funded DPP showed that it was possible to prevent diabetes, the interventions reviewed in this article serve as examples of translating and adapting research into real-world settings in AI/AN communities. These interventions illustrate the need to adapt the diabetes prevention strategies and concepts to the local context to create better chances for positive outcomes. These examples also demonstrate the effectiveness of framing interventions to consider, and adapt from, lessons from epidemiologic, historical, and social ecological contexts to produce outcomes that stem the tide of diabetes in AI/AN communities. The most recent reauthorization of the Congressional funding for SDPI through 2024 will provide more opportunities to further evaluate the program and identify strategies for scale-up and sustainment of these efforts for the future.

The efforts of the IHS to implement a comprehensive diabetes education and treatment program through the SDPI have clearly helped make strides in the prevention and treatment of diabetes in AI/ANs. The SDPI gives hope that research findings can be successfully translated into diverse communities, with attention to the many strategies and social ecological factors that may help or hinder progress.

## Figures and Tables

**Table 1:** Increases in diabetes services provided by the Special Diabetes Program for Indians (SDPI) from 1997 to 2019

Diabetes services	1997	2019
Diabetes clinical teams	30%	95%
Diabetes patient registries	34%	96%
Access to registered dietitians	37%	85%
Access to physical activity specialists	8%	84%
Access to culturally tailored diabetes education materials	36%	96%
Adult weight management services	19%	76%

Adapted from table 1 in the 2020 SDPI 2020 Report to Congress (20); data from the 2019 SDPI grant program evaluation.

## Abbreviations

Indian Health Service (IHS): an agency within the US Department of Health and Human Services and the principal federal health care provider for American Indian and Alaska Native people. Organized into 12 service areas that provide health care services to ~2.6 million AI/AN individuals
A1C: glycated hemoglobin
